# Physical Activity, Sleep, and Function in Assisted Living Residents With Differing Mobility Status

**DOI:** 10.1093/geroni/igaf122.2747

**Published:** 2025-12-31

**Authors:** Katelyn Webster-Dekker, Eileen Hacker, Yvonne Lu

**Affiliations:** University of Michigan School of Nursing, Ypsilanti, Michigan, United States; The University of Texas MD Anderson Cancer Center, Houston, Texas, United States; Indiana University School of Nursing, Indianapolis, Indiana, United States

## Abstract

Physical activity (PA) is crucial for promoting health among older adults. However, those with mobility disabilities are often excluded from PA research. Our study aimed to better understand the relationship between PA and health-related characteristics in older adults in assisted living facilities with different levels of mobility. We compared those who primarily ambulated to those who primarily used a wheelchair or scooter. We recruited 35 participants aged 55 and older from five assisted living facilities. Participants wore an Actiwatch accelerometer for 7 days to monitor PA and sleep and completed physical function assessments. We used Spearman’s correlations to analyze the relationships between PA, sleep, and physical function. We report correlations >0.30 rather than p-values due to the small sample size. Participants had a mean age of 75.3 (SD = 10.6) and were 86% female; 60% primarily ambulated (independently or with an assistive device), while 40% used wheelchairs or scooters. Overall, PA volume was negatively correlated with sleep time (r=-0.38). Among ambulatory residents, poorer sleep quality (increased wake after sleep onset and number of awakenings) correlated with less PA (r=-0.46 and -0.32). In participants using wheelchairs or scooters, PA showed positive correlations with handgrip strength (r = 0.47) and short physical performance battery total score (r = 0.48). In all participants, balance positively correlated with PA (r = 0.37). These findings underscore the interplay between mobility status, PA, sleep, and function in older adults in assisted living, suggesting that tailored interventions are needed to enhance health in these populations.

